# Research of Nanomaterials as Electrodes for Electrochemical Energy Storage

**DOI:** 10.3390/molecules27030837

**Published:** 2022-01-27

**Authors:** Nataliya N. Yazvinskaya, Mikhail S. Lipkin, Nikolay E. Galushkin, Dmitriy N. Galushkin

**Affiliations:** 1 Laboratory of Electrochemical and Hydrogen Energy, Department of Radioelectronic and Electrotechnical Systems and Complexes, Don State Technical University, 346500 Shakhty, Russia; lionnat@mail.ru (N.N.Y.); dmitrigall@yandex.ru (D.N.G.); 2 Department of Chemical Technologies, Platov South-Russian State Polytechnic University, 346428 Novocherkassk, Russia; lipkin@yandex.ru

**Keywords:** hydrogen accumulation, nanomaterial, energy storage

## Abstract

This paper has experimentally proved that hydrogen accumulates in large quantities in metal-ceramic and pocket electrodes of alkaline batteries during their operation. Hydrogen accumulates in the electrodes in an atomic form. After the release of hydrogen from the electrodes, a powerful exothermic reaction of atomic hydrogen recombination with a large energy release occurs. This exothermic reaction is the cause of thermal runaway in alkaline batteries. For the KSL-15 battery, the gravimetric capacity of sintered nickel matrix of the oxide-nickel electrode, as hydrogen storage, is 20.2 wt%, and cadmium electrode is 11.5 wt%. The stored energy density in the metal-ceramic matrix of the oxide-nickel electrode of the battery KSL-15 is 44 kJ/g, and in the cadmium electrode it is 25 kJ/g. The similar values for the KPL-14 battery are as follows. The gravimetric capacity of the active substance of the pocket oxide-nickel electrode, as a hydrogen storage, is 22 wt%, and the cadmium electrode is 16.9 wt%. The density of the stored energy in the active substance oxide-nickel electrode is 48 kJ/g, and in the active substance of the cadmium electrode it is 36.8 kJ/g. The obtained results of the accumulation of hydrogen energy in the electrodes by the electrochemical method are three times higher than any previously obtained results using the traditional thermochemical method.

## 1. Introduction

Finding an optimal method of storing hydrogen in hydrogen power is the most important and technically difficult task [1,2,3,4]. Currently, the best method of storing hydrogen in terms of safety and efficiency is considered to be the use of sorbent materials and metal hydrides [1,2,3,4,5,6,7,8]. The main problem of reversible hydrogen storage is the achievement of optimal indicators for the capacity of accumulated hydrogen. Kinetic and thermodynamic parameters for hydrogen storage systems are also very important. As part of the problem discussed, research has been carried out over the past 10 years on mainly nanostructured carbon materials and complex hydrides of the light elements. Unfortunately, at present, the achieved parameters are small for the practical use of hydrogen storage systems [1,2,3,4,5,6,7,8,9,10,11,12,13,14,15].

The U.S. Department of Energy (DOE) most definitely formed criteria for onboard hydrogen storage systems as part of the country’s hydrogen energy development program. These criteria were developed taking into account the requirements of the U.S. automotive industry [16,17]. The main criterion is the specific capacity of the system. Here, the main indicator is the achievement by 2020 of gravimetric capacity parameters of more than 5.5 wt%, and by a volume capacity of more than 40 kg m^−3^. However, until now, these requirements have not been fulfilled, and despite some changes in these requirements, they remain relevant to this day.

If we consider all simple hydrides, then in terms of volumetric and gravimetric capacity, magnesium hydrides (MgH_2_) and its alloys have the highest values of 110 kg m^−3^ and 7.66 wt%, respectively. From the point of view of theoretical parameters, the capacity of some complex hydrides can exceed the value of the capacity of magnesium hydride [1]. However, if we consider this issue by taking the reversibility of the system into account, then the reversible capacity of complex hydrides is not greater than the capacity of magnesium hydrides [2].

Unfortunately, despite the large amount of work done, the researchers failed to develop an optimal onboard hydrogen storage system that meets the criteria formulated by the U.S. Department of Energy for the automotive industry. In this regard, it has not yet been possible to create onboard hydrogen storage systems with the necessary parameters for the automotive industry, which is an obstacle to the widespread introduction of hydrogen energy into the global economy.

Currently, three technologies for the production of metal hydrides are used in the world.

1. Chemical method of producing hydrides. Here, metal hydrides are synthesized by chemical interactions [1], but this can be realized only for a limited amount of hydrides [18]. The disadvantage of this method of producing hydrides is the irreversibility of this process. Fundamentally, the process of regenerating the starting hydrides for their use in chemical reactions is possible, but not practical, based on economic considerations. Because of this, the method discussed is considered unacceptable for onboard hydrogen storage systems.
2. The standard thermochemical method allows the accumulation of hydrogen in metal hydrides and carbon materials by applying a certain temperature and pressure for hydrogenation (T1, P1) and dehydrogenation (T2, P2), respectively:
  (1) $${\mathrm{CMH}}_{n}\overset{P_{1},T_{1}}{\leftarrow }\overset{P_{2},T_{2}}{\to }\mathrm{CM}+\frac{n}{2}H_{2},$$
  where CM is carbon material or metal.
3. The electrochemical method is associated with the accumulation of hydrogen in metal hydrides and carbon materials by decomposition of the electrolyte. Hydrogen, which is released as a result of the reaction, is adsorbed at the cathode. Then most of it escapes into the atmosphere, and a small part of hydrogen penetrates into the deep layers of the cathode. In addition, due to the dense packing of the electrodes in batteries, part of the hydrogen enters the anode and accumulates in it. First, hydrogen is present inside the cathode (and anode) in an unbound state as the α-phase. With a long electrolysis process, hydrogen forms bound states over time with the metal in the form of a β-phase. Metal hydrides [1,18] are thus formed.

During battery charging, hydrides are formed only in the thin surface layer of the electrodes [19] and the gravimetric capacity of the electrodes increases insignificantly.

The considered method of hydrogen accumulation into the electrodes is not new [1]; however, the electro-chemical method has never been used for hydrogen accumulation in hydrogen storage systems.

Nevertheless, with our studies of thermal runaway in alkaline accumulators [19,20,21,22,23,24], and by obtaining positive results in this direction, it was possible to prove the effectiveness of the above method for hydrogen accumulation [25,26,27].

An effect can occur when charging some types of nickel-cadmium accumulators at a constant voltage, as well as during the floating charge process, called the thermal runaway [19,20].

When this effect occurs in the battery, the charge current increases sharply, and this leads to evaporation of the electrolyte. Sometimes there were cases of fire and explosion of the battery or melting of the plastic body [19,20,21,22,23,24].

A number of works [20,25,27] researching the process of thermal runaway on the basis of alkaline batteries of KSX-25 with a capacity of 25 of Ah received results of experimental studies which showed that at thermal runaway, there is a release of the gas mix consisting of approximately 284 L of hydrogen and 13 L of oxygen.

Based on these data, it can be assumed that hydrogen accumulates in the electrodes of the batteries as their useful life increases, and accordingly, the longer this time, the more hydrogen can accumulate in them.

If we accept this assumption as the main hypothesis, then the amount of hydrogen accumulated in the electrodes of the batteries will be determined primarily by their service life at the facility. In this paper, this hypothesis will be tested.

## 2. Results

In order to carry out experimental studies on the above-mentioned problems, KSL-15 batteries with a capacity of 15 Ah were used as the object of the study. These batteries had sintered positive and negative electrodes. The period of operation of the considered batteries was in the range from 0 to 7.5 years. In addition, in experimental studies, KPL-14 batteries with a capacity of 14 Ah with pocket electrodes were used. These batteries had a service life in the range of 0 to 10 years. The experimental part of the study was carried out on a laboratory bench designed to control the gas release process during the thermal decomposition of electrodes from various batteries.

**Figure 1:**
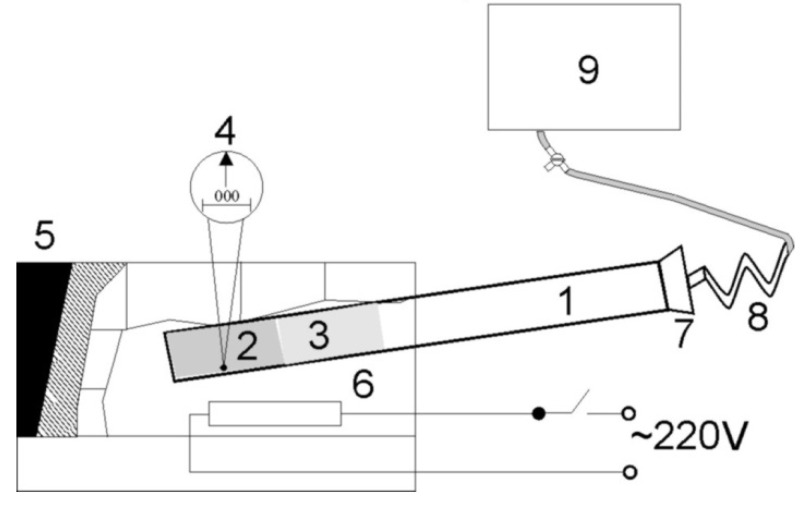
Installation scheme for thermal decomposition of electrodes: (1) heat chamber, (2) cartridge, (3) porous ceramic plug, (4) thermocouple, (5) muffle furnace, (6) heater, (7) rubber plug with a tube for gas extraction, (8) standard coil, and (9) measuring container for released gas.

For more information on the work of the laboratory stand, refer to the Ref. [26] and (Figure 1).

The laboratory stand (Figure 1) consists of a metal chamber 1, where the electrode for thermal decomposition is placed. The chamber has a cylindrical shape. The length of the chamber is 1.8 m and 2 cm in diameter. On one side, the chamber is sealed. The sealed side of the metal chamber was placed for heating in the muffle furnace. The other side of the metal chamber was connected to the measuring container (9) through a rubber plug (7) with a tube (8) for discharging the released gas mixture.

At the next stage of the experiment, the electrode was heated on a laboratory bench in a thermal chamber. The gas mixture released during heating was cooled by a standard coil when passing through it into a measuring container.

The electrode in the thermal chamber decomposed at a high temperature and adhered to the walls of the thermal chamber. In order to avoid this problem, the electrode was placed in a special cartridge (2). Each electrode was folded into a tube and placed in a cartridge prior to thermal decomposition. A porous ceramic plug (3) (20 cm) was inserted above the cartridge to suppress convection in the heat chamber.

Each electrode in the thermal chamber was decomposed under the influence of a temperature of 800 °C. The choice of such a decomposition temperature of the electrodes was not random. In a number of our studies on a thermal runaway, it was found that the gas release during thermal decomposition of the cadmium electrode begins at a temperature of 340 °C. Gas release during thermal decomposition of the oxide-nickel electrode begins at a temperature of 660 °C. Intensive gas release during decomposition of the same electrodes begins at a temperature of more than 740 °C. In accordance with this data, the operating temperature value for decomposition of batteries electrodes was chosen to be 800 °C. This value was used as the optimal temperature of thermal decomposition for cadmium and oxide-nickel electrodes.

The thermal decomposition of the electrodes was stopped at release of less than 100 mL gas from one electrode in one day of the experiment. One day of the experiment was 11 h of continuous thermal decomposition of the electrode. For the KSL-15 battery with a service life of more than 5 years, the process of thermal decomposition of cadmium electrodes took an average of 12 days, and for oxide-nickel electrodes, an average of 14 days.

Every day, during the experiment on the thermal decomposition of the cadmium electrode 3.5 L of the gas mixture was released from it at the initial stage, and at least up to 100–110 mL of the gas mixture at the final stage. The daily volume of gas release from the oxide-nickel electrode was at least 5.5 L during the initial period of experimental studies, and at least 100–110 mL of the gas mixture at the final stage.

The obtained results from experimental studies on the basis of KSL-15 batteries are presented in Table 1, and on the basis of KPL-14 batteries, are presented in Table 2.

Table 1 and Table 2 show the average values of hydrogen evolved from three electrodes (separately for oxide-nickel and cadmium electrodes) from each tested battery.

It should be noted that when an empty heat chamber is heated, about 180–190 mL of gas flows into the measuring container, due to the fact that when the installation is heated, the air in the sealed chamber expands. Therefore, when measuring the gas evolved from the electrode, we subtracted 190 mL of gas.

Obtained as a result of experimental studies on thermal decomposition of electrodes of different types, the gas mixture was analyzed by a gas analyzer VOG-2M with absolute error in percentage concentrations of 0.3–0.5. It has been found that the gas mixture consists entirely of hydrogen.

## 3. Discussion

From the results of the conducted experiments (Table 1 and Table 2), it can be seen that the amount of hydrogen released from the electrodes increases with increasing battery life. It is also worth noting that during the thermal decomposition, hydrogen is not released from electrodes and new and non-previously operated accumulators.

It is also worth noting that with a short battery life, the amount of hydrogen accumulated in their electrodes varies quite significantly from battery to battery; this can presumably be due to different operating conditions of the batteries. With a rather long battery life of more than five years, the amount of hydrogen that accumulated in the electrodes is, on average, approximately the same. In batteries with long service lives, a large amount of hydrogen can accumulate. So, for example, in the battery, KSL-15 contains five cadmium and six oxide-nickel electrodes. From this, it can be found that in one battery, KSL-15 (No. 6, Table 1), the total amount of accumulated hydrogen is approximately 291 L. For a battery, KPL-14, with four cadmium and five oxide-nickel electrodes in its composition (No. 5, Table 2), the hydrogen content is approximately 289.4 L.

We consider calculations showing that during the operation of the batteries, it is possible to accumulate such a volume of hydrogen in electrodes.

From the maintenance manual of KSL-15 batteries, one can see that they are charged with a current of 3.8 A for 6 h. Therefore, to fully charge these batteries, they must be recharged 1.52 times their nominal capacity. Thus, when charging batteries of this brand, almost 7.8 Ah is spent on the decomposition of electrolyte into hydrogen and oxygen. Hence, during the experiment, during one cycle of charge for a KSL-15 battery, up to 3 L of hydrogen and 1.5 L of oxygen were released. Therefore, in theory, it can be assumed that the amount of hydrogen equal to 291 L stored in the electrodes of the batteries KSL-15 can be accumulated over 97 charge-discharge cycles. As a rule, in practice, during long-term operation of the battery (more than five years), the number of charge-discharge cycles can be tens of times more, and therefore, the studied battery can accumulate the amount of hydrogen obtained in the experiment.

If we consider a battery, KPL-14, which is charged with a current of 2.5 A for 10 h, it can also be argued here that the accumulated amount of hydrogen in the electrodes of this battery can theoretically be accumulated at less than 66 charge-discharge cycles. During their operation (over five years), KPL-14 batteries go through at least 10 times more charge-discharge cycles. Therefore, they can also accumulate in their electrodes the amount of hydrogen found in experiments (Table 2).

As is known, when charging nickel-cadmium batteries with direct current, electrolyte decomposes into hydrogen and oxygen, but the experiments conducted (Table 1 and Table 2) show that oxygen does not accumulate in electrodes, only hydrogen. Moreover, hydrogen accumulates both in oxide-nickel and cadmium electrodes in very large quantities. This is due to the fact that hydrogen has very high diffusion permeability. At a temperature of 20 °C, the diffusion coefficient of hydrogen in nickel is about 10^10^ times greater than nitrogen or oxygen [1].

Now we will evaluate the parameters of the sintered nickel matrix of the oxide-nickel electrode as a hydrogen accumulator. According to the experimental data (No. 6, Table 1), over a long period of operation of KSL-15 batteries, about 32 L of hydrogen can accumulate in the sintered matrix of an oxide-nickel electrode. The weight of the oxide-nickel electrode is 21.3 g, including a mass of the active substance of 7.15 g (Ni(OH)_2_) and the mass of the sintered nickel matrix of 14.15 g. Hence, the gravimetric capacity of an oxide-nickel electrode is 13.4 wt%. Hydrogen accumulates in the sintered nickel matrix of an oxide-nickel electrode [25]. Then the gravimetric capacity of the sintered nickel matrix of an oxide-nickel electrode is 20.2 wt%.

The mass of the sintered cadmium electrodes is 16.3 g. Thus, the gravimetric capacity of the cadmium electrode is 11.5 wt%.

Similar values for a KPL-14 battery are as follows. The active substance mass of an oxide-nickel electrode is 14.7 g (2% BaSO_4_, 20% graphite powder, the rest Ni(OH)_2_) and the gravimetric capacity is 22 wt%. The mass of the active substance of the cadmium electrode is 14.3 g (CdO), and the gravimetric capacity is 16.9 wt%.

The data obtained as a result of this study exceed all the previously obtained results by four times using the traditional thermochemical method (1) for any previously considered reversible metal hydrides, including magnesium hydride or complex hydrides [1,2].

The study of the reasons for such a large accumulation of hydrogen in the electrodes of batteries using the electrochemical method is of great importance for the development of hydrogen energy. However, these studies are now only in the early stages.

In some studies [28,29], it was shown that grinding a metal powder leads to a sharp increase in the accumulation of hydrogen in it.

Sintered electrode matrices of batteries are made of finely dispersed nickel powder by sintering it.

Therefore, first of all, we studied the effect of the porosity of the electrodes on the gravimetric capacity of the sintered electrodes. For this, several oxide-nickel electrodes of the KSL-15 battery were replaced by compact nickel electrodes of the same area and mass. Then the KSL-15 battery was charged with a current of 1 A for a year. After that, the electrodes were subjected to thermal decomposition in the installation (Figure 1) to determine their gravimetric capacity. The gravimetric capacity of the compact nickel electrodes turned out to be 0.9 wt%, while for cermet nickel matrices of oxide-nickel electrodes it, as usual, was equal to 20.2 wt%. Thus, the porous structure of the sintered electrodes significantly increases their gravimetric capacity.

The sintered electrode is a metal made of deeply destructed crystal structures. However, any imperfections in the metal crystalline structure are traps for hydrogen, as they decrease the energy of the hydrogen atom as compared to their location in normal interstice. Besides, they are the centers of hydrogen absorption, and also contribute to hydrogen penetration into the metal depth.

In addition, we showed (using the Scanning Electron Microscopy and the Transmission Electron Microscopy) that in the electrodes, where the microdefects and dislocations density was greater, the gravimetric capacity of these electrodes was also greater; although, of course, it is still necessary to investigate the influence of different types of microdefects on the accumulation of hydrogen in electrodes.

In a few papers [30,31], it was shown that oxides of transition metals are catalysts for the accumulation of hydrogen.

Therefore, in another series of experiments, we studied the effect oxides of transition metals on the gravimetric capacity of the sintered electrodes. For this, several oxide-nickel electrodes of the KSL-15 battery were replaced by sintered nickel matrices of these electrodes (without nickel hydroxide). Then the KSL-15 battery was charged with a current of 1 A for a year. After that, the electrodes were subjected to thermal decomposition in the installation (Figure 1) to determine their gravimetric capacity. The gravimetric capacity of the sintered nickel matrices of these electrodes (without nickel hydroxide) was 13 wt%, while for cermet nickel matrices of oxide-nickel electrodes, it, as usual, was equal to 20.2 wt%. These experiments proved that nickel hydroxide is a powerful catalyst for the accumulation of hydrogen in porous metals.

Undoubtedly, the long duration of the accumulation of hydrogen during the electrochemical decomposition of the electrolyte contributes to the accumulation of hydrogen in the electrodes of the batteries in large quantities.

However, the phenomenon of accumulation of very large amounts of hydrogen in the electrodes of batteries, like any new phenomenon, gives rise to many problems that need to be further investigated. The main of these problems are follows.

Firstly, it is necessary to investigate the influence of various metals (and active materials) on the gravimetric capacity of hydrogen storage in electrodes using the electrochemical method of hydrogen accumulation in electrodes.

Secondly, it is necessary to investigate the reasons for the accumulation of hydrogen in such large quantities in the electrodes of batteries by the electrochemical method in detail.

Thirdly, it is necessary to investigate the structure of the resulting metal hydrides using X-ray diffraction (XRD) and scanning electron microscope (SEM), and so forth.

To solve these problems, large-scale and long-term research is required, which we are currently conducting. Therefore, solving these problems is the topic of our future publications. Hydrogen is present in the electrodes of the KSL-15 battery in the form of metal hydrides, that is, in atomic form [1], and after its release from the electrodes it recombines:

H + H → H_2._ (2)

The reaction (2) is an exothermic reaction with heat release of 436 kJ/mol (hydrogen) [32]. The heat release of reaction (2) is greater than the heat release during the combustion reaction of hydrogen in oxygen 285.8 kJ/mol (hydrogen) [33,34].

Now, knowing the numerical values of the gravimetric capacity and hydrogen recombination energy, we can calculate the stored energy density in the metal-ceramic matrix of the oxide-nickel electrode of the battery KSL-15, which is 44 kJ/g, and in the cadmium electrode the density is stored energy 25 kJ/g. The similar values for the KPL-14 battery are as follows. The density of the stored energy in the active substance oxide-nickel electrode is 48 kJ/g, and in the active substance of the cadmium electrode is 36.8 kJ/g.

## 4. Conclusions

Hydrogen energy is accumulated in the nanomaterials of the electrodes of nickel-cadmium batteries in large quantities. The sintered matrix of oxide-nickel electrodes is made by sintering nickel nano-powder in an inert atmosphere. In pocket oxide-nickel electrodes, hydrogen is accumulated in carbon nano-powders, which are an electrically conductive additive in these electrodes. In pocket cadmium electrodes, hydrogen is accumulated in cadmium nano-powder, which is formed during the accumulation of hydrogen in these electrodes and partial reduction of cadmium oxides.

The phenomenon of hydrogen accumulation in large quantities in the electrodes of nickel-cadmium batteries has recently been established [35]. Therefore, this phenomenon requires further theoretical and experimental studies.

## Figures and Tables

**Table 1:** Dependence of the amount of accumulated hydrogen in one electrode of the KSL-15 battery (with sintered electrodes) on the battery service life ^1^.

Battery Number	1	2	3	4	5	6	7	8	9
Service life (years)	New	1.0	1.6	3.4	4.2	5.1	5.3	6.2	7.5
Amount of gas (Ni) (liters)	0	11.0	16.3	25.2	28.7	32.0	32.1	32.1	32.0
Amount of gas (Cd) (liters)	0	11.0	12.8	15.4	19.9	21.0	20.7	20.9	21.1

^1^ The relative error of the data in the Table 1 and Table 2 is 5–6%.

**Table 2:** Dependence of the amount of accumulated hydrogen in one electrode of the KPL-14 battery (with pocket electrodes) on the battery service life ^1^.

Battery Number	1	2	3	4	5	6	7	8	9
Service life (years)	New	1.0	2.1	4.4	6.2	7.1	8.3	9.0	10.0
Amount of gas (Ni) (liters)	0	16.4	31.3	35.7	36.2	36.1	36.0	35.9	36.0
Amount of gas (Cd) (liters)	0	8.5	16.3	24.6	27.1	27.2	27.0	27.0	26.9

^1^ The relative error of the data in the Table 1 and Table 2 is 5–6%.

## Data Availability

Not applicable.

## Sample Availability

Samples of the compounds are available from the authors.
